# Supplementary material for: Association between oral microbiome and seven types of cancers in East Asian population: a two-sample Mendelian randomization analysis
Source: Front Mol Biosci. 2023 Nov 21;10:1327893. doi: 10.3389/fmolb.2023.1327893 (PMC10702768; doi:10.3389/fmolb.2023.1327893)
Supplement: Supplementary file 1 [file DataSheet1.ZIP › Supplementary Materials/MR plots of saliva/saliva═╝/colorectal cancer/pheno.3418_to_colorectal cancer_forest.pdf]

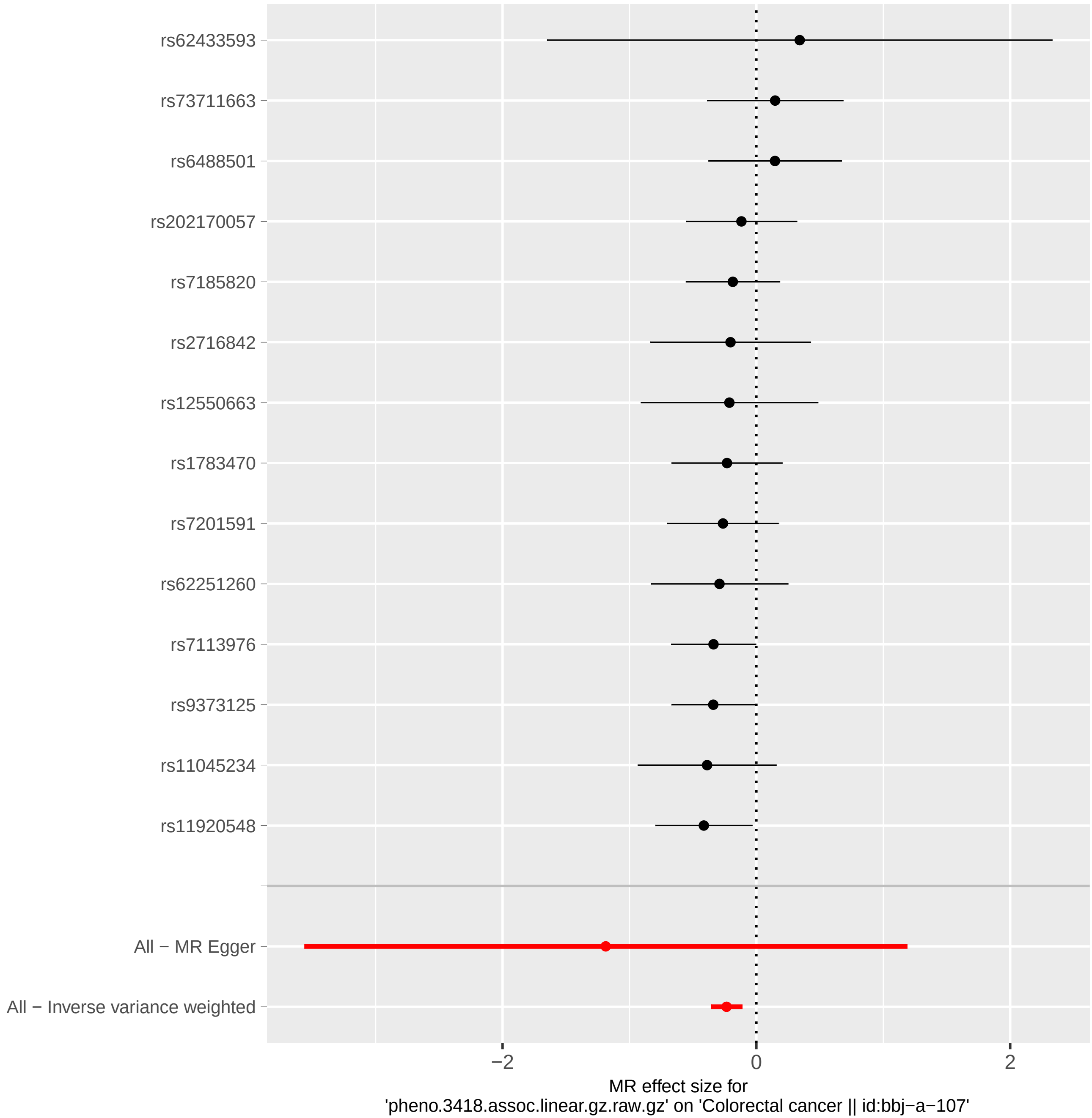
rs62433593
rs73711663
rs6488501
rs202170057
rs7185820
rs2716842
rs12550663
rs1783470
rs7201591
rs62251260
rs7113976
rs9373125
rs11045234
rs11920548
All − MR Egger
All − Inverse variance weighted
−2
0
2
MR effect size for
'pheno.3418.assoc.linear.gz.raw.gz' on 'Colorectal cancer || id:bbj−a−107'
